# Triphenylphosphonium Moiety Modulates Proteolytic Stability and Potentiates Neuroprotective Activity of Antioxidant Tetrapeptides *in Vitro*

**DOI:** 10.3389/fphar.2018.00115

**Published:** 2018-02-19

**Authors:** Rezeda A. Akhmadishina, Ruslan Garifullin, Natalia V. Petrova, Marat I. Kamalov, Timur I. Abdullin

**Affiliations:** ^1^Institute of Fundamental Medicine and Biology, Kazan (Volga Region) Federal University, Kazan, Russia; ^2^Institute of Materials Science and Nanotechnology, National Nanotechnology Research Center (UNAM), Bilkent University, Ankara, Turkey

**Keywords:** aromatic-cationic oligopeptides, triphenylphosphonium compounds, antioxidant activity, Fenton reaction, neuronal cells, oxidative damage, cytoprotection, protease stability

## Abstract

Although delocalized lipophilic cations have been identified as effective cellular and mitochondrial carriers for a range of natural and synthetic drug molecules, little is known about their effects on pharmacological properties of peptides. The effect of triphenylphosphonium (TPP) cation on bioactivity of antioxidant tetrapeptides based on the model opioid YRFK motif was studied. Two tetrapeptide variants with L-arginine (YRFK) and D-arginine (YrFK) were synthesized and coupled with carboxyethyl-TPP (TPP-3) and carboxypentyl-TPP (TPP-6) units. The TPP moiety noticeably promoted YRFK cleavage by trypsin, but effectively prevented digestion of more resistant YrFK attributed, respectively, to structure-organizing and shielding effects of the TPP cation on conformational variants of the tetrapeptide motif. The TPP moiety enhanced radical scavenging activity of the modified YRFK in a model Fenton-like reaction, whereas decreased reactivity was revealed for both YrFK and its TPP derivative. The starting motifs and modified oligopeptides, especially the TPP-6 derivatives, suppressed acute oxidative stress in neuronal PC-12 cells during a brief exposure similarly with glutathione. The effect of oligopeptides was compared upon culturing of PC-12 cells with CoCl_2_, L-glutamic acid, or menadione to mimic physiologically relevant oxidative states. The cytoprotective activity of oligopeptides significantly depended on the type of oxidative factor, order of treatment and peptide structure. Pronounced cell-protective effect was established for the TPP-modified oligopeptides, which surpassed that of the unmodified motifs. The protease-resistant TPP-modified YrFK showed the highest activity when administered 24 h prior to the cell damage. Our results suggest that the TPP cation can be used as a modifier for small therapeutic peptides to improve their pharmacokinetic and pharmacological properties.

## Introduction

Bioactive peptides are a diverse class of biomolecules with multiple functions in living organisms and great therapeutic potential. The peptides are characterized by intrinsically high selectivity, low tissue accumulation and toxicity, and, in comparison with macromolecular biologics, increased physicochemical stability and penetration ability (Vlieghe et al., 2010; Fosgerau and Hoffmann, 2015). Since as few as di- and tripeptide motifs are capable of displaying a relatively specific bioactivity (Guiotto et al., 2005; Ung and Winkler, 2011; Perazzo et al., 2017), the oligopeptides which are commonly composed of fewer than 15 amino acid residues (Sewald and Jakubke, 2015) may be considered a powerful ‘platform’ for development of therapeutics with a range of activities.

Bioactive oligopeptides are being discovered among different biological species, tissues and protein resources. Their specific functions encompass at least neuro- and immunoregulatory, regenerative, anticoagulant, antihypertensive, anti-inflammatory, antioxidant, antimicrobial and anticancer activities [see reviews (Guiotto et al., 2005; Anisimov and Khavinson, 2010; Kim and Wijesekara, 2010; Sah et al., 2015; Yoshikawa, 2015)]. An important manifestation of many small peptides relates to their antioxidant properties, which are of particular interest in regulation of pathological production of reactive oxygen species (ROS) (Kohen and Nyska, 2002; Pham-Huy et al., 2008). Oligopeptides are relevant candidates for selective and safe antioxidants targeted at treatment and/or prevention of ROS-associated inflammatory, cancer and degenerative diseases.

A fundamental strategy for generation of therapeutic oligopeptides, including antioxidant ones, relies on reproduction of natural peptide structures followed by their consecutive chemical modification and introduction of amino acid analogs in order to overcome intense biodegradation, relatively low tissue and cellular availability of peptide molecules (Witt et al., 2001; Adessi and Soto, 2002; Vlieghe et al., 2010). The tripeptide glutathione (γ-Glu-Cys-Gly, GSH) and histidine-containing dipeptides, namely carnosine (β-Ala-His) and its homologs, are predominant in mammals and the most studied oligopeptides with intrinsic ROS scavenging and antioxidative functions (Schafer and Buettner, 2001; Guiotto et al., 2005). Administration of these oligopeptides and their esters was shown to provide antiradical, cyto- and tissue-protecting, immune-modulating, anticancer and antiviral effects *in vitro* and *in vivo* (Lomaestro and Malone, 1995; Guiotto et al., 2005; Fraternale et al., 2009; Nichols et al., 2012; Zampagni et al., 2012).

Main chemical approaches to synthesis of carnosine derivatives with improved pharmacokinetic properties are generally based on modifications of the carboxyl and amino groups of the dipeptide and/or replacement of L-histidine with its D-enantiomer (Bellia et al., 2012). Similarly, derivatization of GSH with aliphatic, aromatic or cationic moieties via thiol or terminal reactive groups of the tripeptide was reported to generate enhanced antioxidants with antiviral and neuroprotective activities (Sheu et al., 2006; Fraternale et al., 2009; Nichols et al., 2012; Zampagni et al., 2012).

Bioinspired artificial tetrapeptides comprising alternating aromatic (tyrosine, phenylalanine) and cationic (arginine, lysine) amino acids, called Szeto–Schiller (SS) peptides, were earlier developed as dermorphin analogs with pronounced scavenging action against different ROS (Schiller et al., 1989; Szeto, 2006a). The aromatic-cationic structure of these peptides, i.e., Tyr-^D^Arg-Phe-Lys-NH_2_ (starting sequence) and ^D^Arg-Dmt-Lys-Phe-NH_2_ (SS-31 sequence), allows them to pass the plasma membrane and concentrate at the inner membrane of mitochondria (Szeto, 2006a). Cytoprotective and antiapoptotic effects of the SS-peptides were shown to provide therapeutic benefit, for instance, upon exposure to cell-damaging agents (Zhao et al., 2005), ischemia-reperfusion injury (Szeto, 2008), hypertensive cardiomyopathy (Dai et al., 2011), neurodegeneration (Cho et al., 2007).

Other oligopeptide structures, similar to SS-peptides, with tunable cationic-lipophilic balance were synthesized by using phenylalanine and its artificial analog cyclohexylalanine as a hydrophobic unit (Horton et al., 2008; Yousif et al., 2009). Arginine-related bicyclic guanidinium oligomer, which is characterized by enhanced intracellular and intramitochondrial accumulation, was previously developed (Fernandez-Carneado et al., 2005; Yousif et al., 2009). Recently, a series of D-tetrapeptides containing two tyrosine residues were synthesized, and N-terminally trifluoroacetylated Tyr-Tyr-His-Pro-HN_2_ and Tyr-Tyr-Pro-His-NH_2_ sequence motifs were shown to have highest ability to scavenge ROS and inhibit membrane lipid peroxidation, which was comparable to that of Trolox (Sandomenico et al., 2017).

Among natural and artificial modifiers of bioactive peptides, phosphorus-containing compounds can be used to modulate physicochemical, pharmacokinetic and pharmacological properties of oligopeptides. As shown recently, dithiophosphoric acids form stable ammonium salts with GSH which possess enhanced cellular availability and antioxidant properties compared with the unmodified tripeptide (Akhmadishina et al., 2017). Triphenylphosphonium (TPP) compounds are delocalized lipophilic cations (DLC) which effectively deliver small drugs into mitochondria. The principles of TPP-assisted drug transport across plasma and mitochondrial membranes as well as examples of proposed TPP based therapeutics are reviewed in (Murphy and Smith, 2007; Zielonka et al., 2017). These therapeutics, exemplified by TPP derivatives of vitamin E, coenzyme Q10, TEMPOL, antioxidant enzyme mimetics, have been studied as mitochondria-targeted antioxidants with great potential in treating tissue damage and degeneration (see Murphy and Smith, 2007 and references within).

Furthermore, conjugation of the TPP cations to various biomolecules, including vitamins, terpenes/terpenoids, polyphenols, antibiotics was also proved to be a promising strategy for generation of effective antimicrobial and anticancer compounds (Pugachev et al., 2013; Strobykina et al., 2015; Tsepaeva et al., 2017; Zielonka et al., 2017). In spite of established importance of the TPP cations as a carrier and pharmacophoric groups, little is known about their effects on the properties of therapeutic peptides. As shown in Ross et al. (2004), conjugation of the TPP moiety to penetratin (16-mer) and Tat (12-mer) cell-penetrating peptides does not provide these peptides with the ability to pass inner mitochondrial membrane, whilst attachment of up to three TPP groups to hemagglutinin A derivatives (11–13-mers) considerably enhances their accumulation in mitochondrial matrix (Abu-Gosh et al., 2009). There is, however, the lack of data concerning activity and structure related properties of oligopeptides modified with TPP compounds.

In our study, we focus for the first time on the effect of TPP cation on antioxidant and neuroprotective properties of model therapeutic oligopeptides which relate to the SS-peptides. For this purpose, carboxyethyl-TPP (TPP-3) and carboxypentyl-TPP (TPP-6) were conjugated to Tyr-Arg-Phe-Lys-NH_2_ (YRFK-NH_2_) based tetrapeptide containing arginine residue in its L- or D-form.

## Materials and Methods

### Materials

4-(2′,4′-Dimethoxyphenyl-Fmoc-aminmethyl)-phenoxyacetamido-methylbenzhydryl amine resin (Rink amide MBHA resin), Fmoc-Tyr(tBu)-OH, Fmoc-Arg(Pbf)-OH, Fmoc-D-Arg (Pbf)-OH, Fmoc-Phe-OH, Fmoc-Lys(Boc)-OH, (4-carboxypentyl)triphenylphosphonium bromide, (2-carboxyethyl)triphenylphosphonium bromide, 2-(1H-benzotriazol-1-yl)-1,1, 3,3-tetramethyluronium hexafluorophosphate (HBTU), N,N-diisopropylethylamine (DIPEA), triisopropylsilane (TIPS), trifluoroacetic acid (TFA), N,N-dimethylformamide (DMF), dichloromethane (DCM) were purchased from Merck and Fisher Scientific.

2′,7′-dichlorofluorescin diacetate (DCFDA), 3-(4,5-dimethyl-thiazol-2-yl)-2,5-diphenyltetrazolium bromide (MTT), 2,2′ -diphenyl-1-picrylhydrazyl (DPPH) were purchased from Sigma–Aldrich. 3-(4,5-dimethylthiazol-2-yl)-5-(3-carboxymethoxyphenyl)-2-(4-sulfophenyl)-2H-tetrazolium, inner salt (MTS) were purchased from Promega. Reduced glutathione (purity 98%), L-glutamic acid, menadione, cobalt (II) chloride were produced by Acros Organics. Hydrogen peroxide (30%) was purchased from TatKhimProduct company (Russia). Materials for cell culturing and trypsin from porcine pancreas were obtained from PanEco company (Russia).

LC-MS grade acetonitrile 99.9%, formic acid 99.5% (Fisher Scientific) and trifluoroacetic acid 99.0% (Sigma–Aldrich) were used. Milli-Q grade water (Milli-Q Advantage A10, Merck Millipore) was used to prepare buffers and solutions.

### Synthesis of Oligopeptides

The oligopeptides (YRFK-NH_2_, YrFK-NH_2_, TPP-3-YRFK-NH_2_, TPP-6-YRFK-NH_2_, and TPP-6-YrFK-NH_2_) were synthesized using solid phase peptide synthesis (SPPS) method. The synthesis was performed on a programmable microwave peptide synthesizer Initiator+ SP Wave (Biotage). Rink amide MBHA resin was used as a solid support to obtain peptides with C-terminal amide group. Rink amide resin was swelled by passing DMF and deprotected using 20% (v/v) piperidine solution in DMF. Subsequent iterative coupling and deprotection cycles were carried out using (with respect to resin) 2 equivalents of Fmoc-protected amino acid, 1.95 equivalents of HBTU and 3 equivalents of DIPEA in DMF. Following final deprotection, the oligopeptides were cleaved from the resin directly or after modification with (carboxyalkyl)triphenylphosphonium bromide activated in the same manner as amino acids. The oligopeptides were cleaved from the resin by incubating in cleavage cocktail (95% TFA, 2.5% H_2_O, 2.5% TIPS) for 2–3 h. The cleaved peptides were collected in DCM, which was then removed alongside with TFA on a rotary evaporator. Viscous residual material was triturated with ice-cold diethyl ether, and the peptide precipitate was separated from ether by centrifugation. The precipitate was dissolved in ultrapure water and freeze-dried. The oligopeptides were analyzed by means of high resolution time-of-flight mass spectrometry on an Agilent 1200/6530 instrument with electrospray ionization (ESI) source. A ZORBAX 300SB-C18 column was used to gradually elute oligopeptides using acetonitrile (0.1% formic acid)/water (0.1% formic acid) mixed solvent.

### HPLC Analysis of Oligopeptide Cleavage

The reaction mixture containing an oligopeptide (0.5 mg/mL) and trypsin (5 μg/mL) in phosphate buffered saline (PBS, pH 7.4) was incubated for different time intervals, and aliquots of digestion products were collected for reversed-phase high-performance liquid chromatography (HPLC). HPLC analysis was carried out on a Dionex UltiMate 3000 system (Thermo Scientific) using a Kromasil C18 column, 5 μm, 4.6 mm × 150 mm (AkzoNobel). Mobile phase contained (A) acetonitrile with 0.1% trifluoroacetic acid, (B) milli-Q water with 0.1% trifluoroacetic acid. Gradient scheme was as follows, 0–10 min: from 100% A to 100% B, 10–20 min: 100% A to 100% B, 20–25 min: 100% B. Flow rate was 0.5 mL/min; injection volume was 10 μL. The detection was performed at wavelengths of 220 and 260 nm. Chromatographic data were collected and treated with the aid of Chromeleon 6.80 software (Thermo Scientific). Peak height of the uncleaved oligopeptides was measured to calculate remaining concentration of the oligopeptides after tryptic cleavage. Concentration values were presented as mean ± SD (*n* = 3).

### Evaluation of Cytotoxicity of Oligopeptides

PC-12 rat pheochromocytoma cell line (ATTC) and human skin fibroblasts (HSF) were used. HSF were isolated as detailed in (Tsepaeva et al., 2017). The cells were cultured aseptically in α-MEM containing 10% fetal bovine serum (FBS), 2 mM L-glutamine, 100 U/mL penicillin and 100 μg/mL streptomycin at 37°C in humidified air atmosphere with 5% CO_2_.

The cytotoxicity of the oligopeptides was evaluated in the concentration range from 1 μM to 3.2 mM by means of the MTT proliferation assay in 96-well microplates as described in Tsepaeva et al. (2017). The cell viability was presented as a percentage of control cells grown without compounds (100% viability value). Half-maximal inhibitory concentrations (IC_50_) were calculated from cell viability curves using OriginPro 8.0 software.

### Study of Antioxidant Properties of Oligopeptides

#### Reactions with DPPH Radical and Fenton System

Antioxidant properties of the oligopeptides were analyzed in PBS in microplate format on an Infinite M200 PRO microplate analyzer (TECAN). Briefly, DPPH-assay was performed in the reaction of a serially diluted oligopeptide (from ∼3.3 mM to 2 μM) with 0.25 mM DPPH followed by colorimetric detection of unreacted DPPH at a wavelength of 515 nm (Akhmadishina et al., 2017).

Fenton-like reaction was initiated by mixing 0.2 mM cobalt chloride (CoCl_2_) and 22 mM hydrogen peroxide (H_2_O_2_) in PBS and carried out for 60 min in the presence of or without a serially diluted oligopeptide. ROS generated in the Fenton reaction were detected by using DCFDA fluorescent probe (5 μM) at λ_*ex*_ = 488 nm and λ_*em*_ = 535 nm. The effect of oligopeptides was measured as a percentage of ROS generation rate in the control reaction without effectors (100%) (Akhmadishina et al., 2017).

#### Analysis of H_2_O_2_-Induced Oxidative Stress in Cells

PC-12 cells were seeded in a 96-well plate and allowed to form a subconfluent monolayer. The cells were washed with Hank’s balanced salt solution (HBSS), pre-stained with 20 μM DCFDA and rewashed with HBSS two times. The oxidative stress in the stained cells was induced by incubating them in HBSS solution containing 100 mM H_2_O_2_ for 1 h in CO_2_-incubator. The synthesized oligopeptides or glutathione were added to the extracellular solution in the concentration range from 1 to 10 mM in HBSS and incubated for 1 h. The bottom fluorescent signal was acquired from the treated cells on an Infinite M200PRO microplate analyzer (TECAN) at λ_*ex*_ = 488 nm and λ_*em*_ = 535 nm.

### Evaluation of Cytoprotective Properties of Oligopeptides

PC-12 cells were subjected to oxidative damage by CoCl_2_, L-glutamic acid, or menadione and treated with the oligopeptides as follows. Briefly, the pre-grown cells were seeded in a 96-well plate at a density of 60 000 cells per well and cultured overnight in FBS-containing α-MEM. Next day, the medium was replaced by a fresh one without FBS, and further manipulations were performed in FBS-free medium. The oxidative agents and oligopeptides were supplemented to the medium in three ways: (i) the cells were pre-cultured in the presence of oligopeptides for 24 h, then the medium was replaced by a fresh one supplemented with a damaging factor and the cells were further cultured with the factor for 24 h (CoCl_2_, L-glutamic acid) or 3 h (menadione) (‘pretreatment’); (ii) the damaging factor and oligopeptide were added together to cells and cultured for 24 h (CoCl_2_, L-glutamic acid) or 3 h (menadione) followed by medium replacement (‘cotreatment’); (iii) following treatment with menadione, the cells were additionally cultured with the oligopeptides for 24 h (‘posttreatment’). The concentration of compounds was as follows: oligopeptides 5 mM, CoCl_2_ 2.5 mM, L-glutamic acid 50 mM, menadione 25 μM.

Eventually, viability of the treated cells was evaluated with the use of MTS assay (Promega) on an Infinite M200 PRO microplate analyzer (TECAN). The cell viability was presented as a percentage of that of the control cells cultured in the absence of compounds (100% viability).

### Statistical Analysis

Data were presented as mean ± SD. The statistically significant difference was evaluated by Student’s *t*-test with a significance level of *p* < 0.05.

## Results

### Structure of YRFK Tetrapeptides and TPP Derivatives

**FIGURE 1:**
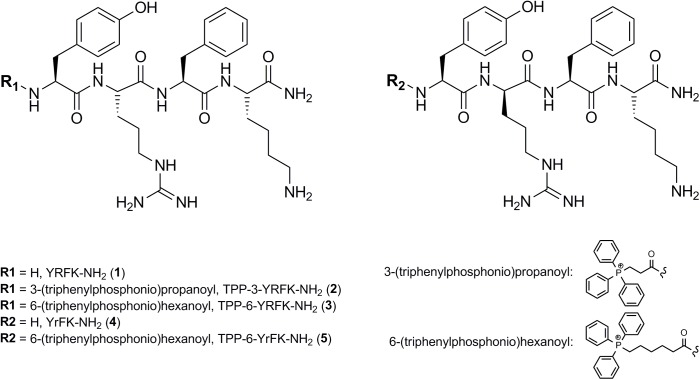
Structure of synthesized YRFK tetrapeptides and their triphenylphosphonium derivatives.

Two starting tetrapeptides based on YRFK sequence motif (**Figure 1**) were synthesized with the use of SPPS technique as detailed in the Section ‘Materials and Methods’. The tetrapeptides were composed of L-tyrosine (Y), L-phenylalanine (F), and L-lysine (K), while varied in arginine enantiomers, i.e., L-arginine (R) or D-arginine (r). The TPP-modified oligopeptides were produced by extending the YRFK motifs with 2-carboxyethyl TPP (TPP-3) or 5-carboxypentyl TPP (TPP-6) units at the N-terminus during the synthesis (**Figure 1**). The primary structure and purity of the YRFK tetrapeptides and their TPP derivatives were verified by LC-MS technique (Supplementary Figure S1).

The carboxyalkyl-TPP unit increased hydrophobicity of the modified oligopeptides in proportion to the length of the aliphatic chain as supported by retention time of the oligopeptides in reversed-phase HPLC (Supplementary Figure S1). According to dynamic light scattering technique, TPP-6-YRFK-NH_2_ and TPP-6-YrFK-NH_2_ formed well-defined submicron aggregates suggesting that the TPP-6 group imparted aggregative properties to the modified oligopeptides (data not shown). The D-arginine-containing variants of oligopeptides (YrFK-NH_2_, TPP-6-YrFK-NH_2_) were characterized by decreased retention time compared with the L-arginine-containing counterparts (Supplementary Figure S1). This indicates altered conformational structure and disturbed hydrophobic properties of the former oligopeptides due to the presence of D-enantiomer of arginine.

### Proteolytic Stability of YRFK Tetrapeptides and TPP Derivatives

Enzymatic cleavage of the oligopeptides was performed in PBS solution using pancreatic trypsin as a pharmacologically relevant endopeptidase (Vlieghe et al., 2010; Danial et al., 2012). To compare proteolytic resistance of the initial and TPP-modified oligopeptides, the reaction conditions were optimized to provide a relatively slow kinetics. The concentration of uncleaved oligopeptides was detected by means of HPLC. In the absence of trypsin, both the unmodified tetrapeptides and TPP derivatives were stable during at least 48 h incubation at ambient temperature.

**FIGURE 2:**
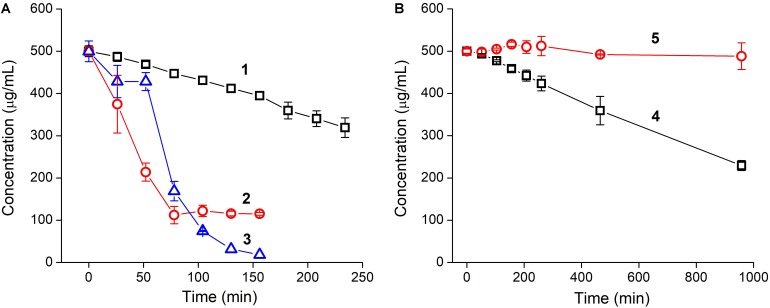
Time kinetics of tryptic cleavage of oligopeptides: **(A)** YRFK-NH_2_ (1), TPP-3-YRFK-NH_2_ (2), TPP-6-YRFK-NH_2_ (3) and **(B)** YrFK-NH_2_ (4), TPP-6-YrFK-NH_2_ (5). Reaction was performed in PBS (pH 7.4) at an initial concentration of oligopeptides of 500 μg/mL. Remaining concentrations of oligopeptides were measured by means of reversed-phase HPLC. Mean ± SD (*n* = 3) are shown.

Incubation of YRFK-NH_2_ with trypsin was accompanied by a gradual depletion of the tetrapeptide (**Figure 2A**, 1), apparently, as a result of cleavage of the peptide bond adjacent to arginine residue (Olsen et al., 2004; Vlieghe et al., 2010). The proteolytic reaction was characterized by linear decrease in YRFK-NH_2_ concentration during 250 min (**Figure 2A**, 1) and afterward (data not shown) with 50% degradation rate (DR_50_) at about 370 min.

**FIGURE 3:**
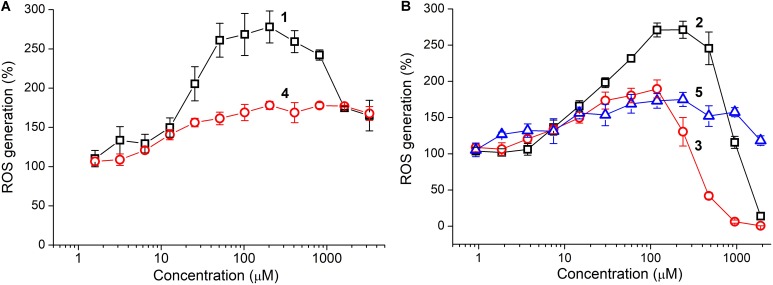
Concentration–dependent effect of oligopeptides on ROS generation in CoCl_2_/H_2_O_2_ reaction: **(A)** YRFK-NH_2_ (1), YrFK-NH_2_ (4) and **(B)** TPP-3-YRFK-NH_2_ (2), TPP-6-YRFK-NH_2_ (3), TPP-6-YrFK-NH_2_ (5). ROS were detected by using DCFDA fluorescent probe (5 μM) at λ_*ex*_ = 488 nm and λ_*em*_ = 535 nm. Mean ± SD (*n* = 3) are shown.

The TPP moiety greatly promoted cleavage of the modified oligopeptides compared with the unmodified motif presumably due to increased affinity of the TPP-containing oligopeptides toward trypsin (see the ‘Discussion’ section). The DR_50_ for TPP-3-YRFK-NH_2_ and TPP-6-YRFK-NH_2_ was observed at about 46 and 70 min, respectively (**Figure 2A**, 2 and 3). Interestingly, the oligopeptides were characterized by different degradation profile. Specifically, TPP-3-YRFK-NH_2_ was rapidly cleaved to the extent of ∼80% followed by a plateau (**Figure 2A**, 2), whereas cleavage of TPP-6-YRFK-NH_2_ was initially retarded during approximately 60 min and then intensified, resulting in rapid and almost complete consumption of the substrate (**Figure 2A**, 3).

**FIGURE 4:**
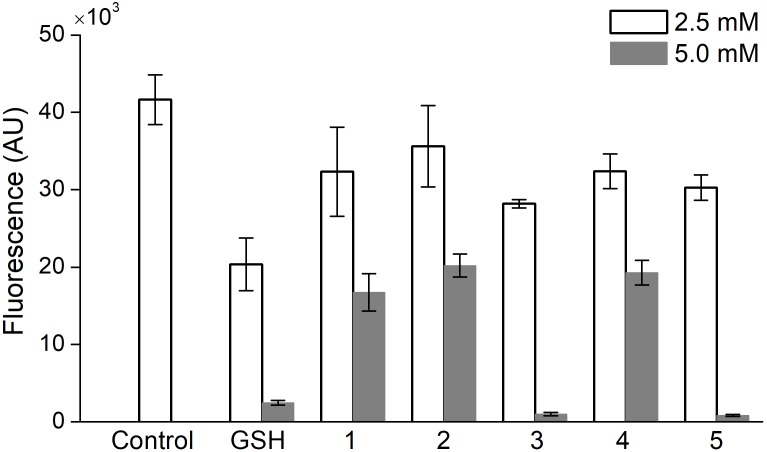
Effect of 1-h incubation with oligopeptides on intracellular ROS level in PC-12 cells treated with H_2_O_2_ (100 mM). Control – uninhibited ROS level, GSH – glutathione, (1) YRFK-NH_2_, (2) TPP-3-YRFK-NH_2_, (3) TPP-6-YRFK-NH_2_, (4) YrFK-NH_2_, (5) TPP-6-YrFK-NH_2_. Analysis was performed in HBSS with the use of DCFDA fluorescent probe. Mean ± SD are shown (*n* = 3).

**FIGURE 5:**
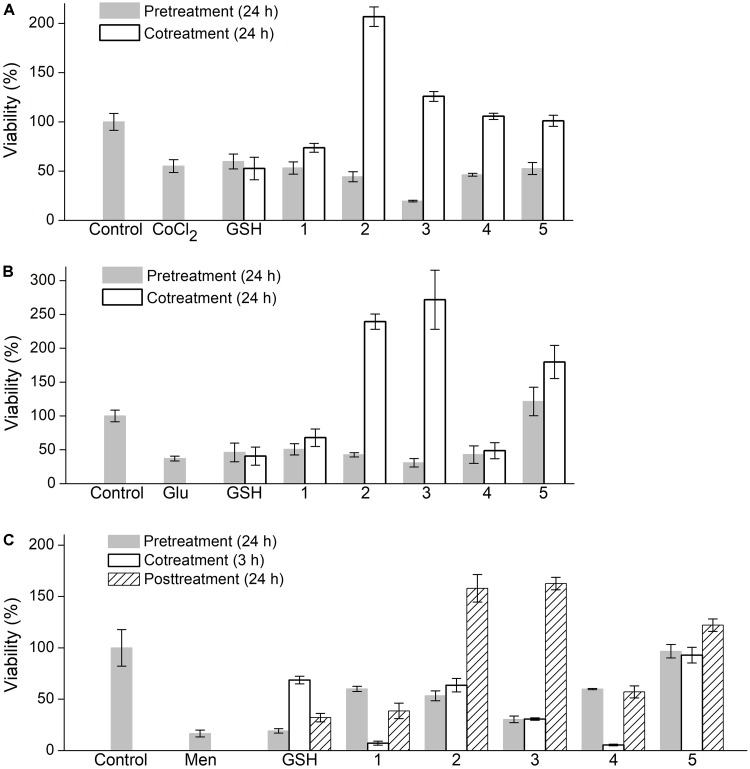
Effect of oligopeptides on viability of PC-12 cells subjected to oxidative factors **(A)** cobalt chloride (CoCl_2_), **(B)** L-glutamic acid (Glu), **(C)** menadione (Men). Concentration of oligopeptides and GSH 5 mM, CoCl_2_ 2.5 mM, Glu 50 mM, Men 25 μM. Oligopeptides were supplemented to medium (FBS-free α-MEM) prior to factors (‘pretreatment’), together with factors (‘cotreatment’) or following factors (‘posttreatment’). Cell viability was evaluated by MTS assay. Mean ± SD are shown (*n* = 3).

In comparison with YRFK-NH_2_, the D-arginine-containing motif was characterized by significantly slower cleavage kinetics with DR_50_ at about 876 min (**Figure 2B**, 4). Under the same conditions, TPP-6-YrFK-NH_2_ did not undergo any degradation during at least 17 h of the reaction (**Figure 2B**, 5), indicating high resistance of this oligopeptide against trypsin in great contrast to the TPP-modified variants which have L-arginine residue (**Figure 2A**, 2 and 3). The results obtained show increased proteolytic stability of YrFK-NH_2_ and especially its TPP derivative, which will be further discussed regarding their conformational features.

### Pro- and Antioxidant Properties of Oligopeptides

Antioxidant activity of the tetrapeptides and TPP derivatives was evaluated by using the colorimetric DPPH assay as well as fluorescent detection of Fenton-like reaction between cobalt chloride (CoCl_2_) and hydrogen peroxide (H_2_O_2_) with the aid of DCFDA fluorescent probe (Akhmadishina et al., 2017). The oligopeptides showed the lack of scavenging activity against the DPPH radical at a concentration as high as 3 mM, however, they strongly affected ROS generation in the CoCl_2_/H_2_O_2_ reaction (**Figure 3**). The unmodified tetrapeptides substantially promoted the reaction at a concentration above 10 μM, presumably, as a result of coordination to the cobalt ions. This prooxidant effect was much more pronounced for YRFK-NH_2_ with a maximum of ∼280% (vs. 180% for YrFK-NH_2_), but decreased at a concentration of the former tetrapeptide over 200 μM (**Figure 3A**, 1). These results suggest that the spatial structure of tetrapeptides conditioned by the presence of L- or D-arginine residue in the peptide sequence noticeably affects their ability to modulate the Fenton reaction.

Introduction of the TPP moiety to YRFK-NH_2_ greatly enhanced antioxidant properties of the corresponding oligopeptides (**Figure 3B**). TPP-3-YRFK-NH_2_ preserved prooxidant activity of the initial motif at submillimolar concentrations, but completely suppressed ROS formation at a concentration of 2 mM (**Figure 3B**, 2). The prooxidant activity of TPP-6-YRFK-NH_2_ was further decreased, and the inhibitory effect of oligopeptide toward the reaction was observed at concentrations > 250 μM (**Figure 3B**, 3). The half-maximal inhibitory concentration (EC_50_) of TPP-6-YRFK-NH_2_ was 500 ± 25 μM, which was comparable to that of GSH in the same reaction (EC_50_ = 544 ± 39 μM) (Akhmadishina et al., 2017). Attachment of the TPP-6 moiety to YrFK-NH_2_ almost did not alter its concentration effect on ROS generation (**Figure 3B**, 5), in great contrast to YRFK-NH_2_, suggesting that the ability of TPP-6 group to modulate antioxidant properties of the oligopeptides depends on the conformation of peptide component.

### Effect of Oligopeptides on Oxidative Stress in PC-12 Cells

A comparative study of the ability of oligopeptides as well as GSH to inhibit the acute production of intracellular ROS was performed on PC-12 cells, which were stained with the DCFDA probe and treated with 100 mM H_2_O_2_ to induce the oxidative stress. At a concentration of 1 or 10 mM the oligopeptides, respectively, did not affect DCFDA fluorescence in the cells or almost completely suppressed the signal (data not shown). Therefore, to compare the effect of oligopeptides on the ROS level in PC-12 cells, their concentration was set at 2.5 and 5.0 mM (**Figure 4**).

The unmodified tetrapeptides and TPP-3-YRFK-NH_2_ (5 mM) partially decreased the DCFDA fluorescence in PC-12 cells by approximately 50% (**Figure 4**, 1, 4, 2), whereas the TPP-6-modified oligopeptides (5 mM) almost completely decreased the signal similarly with GSH (**Figure 4**, 3, 5). These results indicate the ability of both the initial and modified oligopeptides to reduce oxidative stress *in vitro* after short-term treatment, and that this effect is enhanced by the TPP-6 group, presumably, due to better cellular penetration of oligopeptides bearing the elongated alkyl-TPP unit.

### Cytotoxicity of Oligopeptides

An effect of the tetrapeptides and TPP derivatives on viability of PC-12 cells and human skin fibroblasts (HSF) was evaluated using the MTT assay. The oligopeptides studied generally did not cause a dose-dependent decrease in cell viability in the concentration range up to 3.2 mM, indicating the lack of cytotoxic (anti-proliferative) activity (Supplementary Figure S2). A certain cell-modulating effect of the oligopeptides was, however, observed. In the case of HSF, the oligopeptides partially inhibited cell proliferation, which was more obvious for the TPP derivatives. Specifically, TPP-3-YRFK-NH_2_ and TPP-6-YRFK-NH_2_ were found to decrease cell proliferation only at their intermediate concentrations around 100 μM, and this effect disappeared in both lower and higher concentration ranges. Furthermore, both these oligopeptides increased proliferation of PC-12 cells up to 20% over the whole concentration range (Supplementary Figure S2).

Among the oligopeptides studied, only TPP-6-YrFK-NH_2_ displayed the half-maximal inhibition at millimolar concentrations toward HSF proliferation (Supplementary Figure S2). In view of a 3-day span of the MTT assay, this inhibitory activity is probably associated with persistence of the proteolytically stable TPP-6-YrFK-NH_2_ oligopeptide in the culture medium/inside the cells, and hence its increased effect on cell redox metabolism.

### *In Vitro* Neuroprotective Activity of Oligopeptides

PC-12 cells were exposed to three different damaging factors, such as CoCl_2_, L-glutamic acid (^L^Glu) and menadione (Men), which induce different oxidative states in mammalian cells. Factor concentration and time of treatment were preoptimized as detailed in the Section ‘Evaluation of Cytoprotective Properties of Oligopeptides’ to cause moderate decrease in cell viability detected by the MTS assay. The oligopeptides were supplemented in FBS free culture medium in different ways, namely, for 24 h prior to cell exposure to the damaging factor, together with the factor or for 24 h after the exposure followed by cell viability analysis (**Figure 5**).

In the case of CoCl_2_-induced damage, the synthesized oligopeptides (5 mM) when administered in advance did not exhibit a protective effect on PC-12 cells similarly with GSH (**Figure 5A**). During cotreatment with CoCl_2_ the oligopeptides increased cell viability to a different extent unlike GSH, which insignificantly diminished cell damage. The unmodified tetrapeptide YRFK-NH_2_ slightly increased cell viability by a factor of ∼1.3 (to 74% value) compared with the cells treated with CoCl_2_ alone (55% value), whereas the YrFK-NH_2_ tetrapeptide similarly with its TPP derivative restored cell survival to a level of the untreated cells (100% value, *p* < 0.05). Under the same conditions, TPP-3-YRFK-NH_2_ and TPP-6-YRFK-NH_2_ when supplemented simultaneously with CoCl_2_ were found to stimulate cell viability to almost 207 and 126% values, respectively (**Figure 5A**).

Similar changes were revealed for PC-12 cells exposed to 50 mM ^L^Glu. YRFK-NH_2_, YrFK-NH_2_, and GSH showed a relatively low protective effect (up to 67.8% value for YRFK-NH_2_ vs. 37.1% value for control ^L^Glu-treated cells, *p* < 0.05), whereas the TPP-modified oligopeptides, when applied together with ^L^Glu, not only caused cell survival after oxidative damage but also increased cell viability compared with that of the untreated cells (**Figure 5B**). The corresponding viability values were as follows: 239% (TPP-3-YRFK-NH_2_), 272% (TPP-6-YRFK-NH_2_), and 180% (TPP-6-YrFK-NH_2_).

In the case of Men-treated PC-12 cells, GSH increased cell viability from ∼17 to 69% (cotreatment) and 32% values (posttreatment) (*p* < 0.05), but not when applied prior to the treatment (**Figure 5C**). Unlike GSH, the unmodified tetrapeptides YRFK-Am and YrFK-Am similarly to each other protected the cells to viability values in the range from 39 to 60% (*p* < 0.05) only when supplemented prior and after the damaging factor. Much more pronounced ameliorative effect on Men-treated cells was displayed by the TPP-modified oligopeptides with viability values observed as follows (cotreatment/posttreatment): TPP-3-YRFK-NH_2_ (53%/158%), TPP-6-YRFK-NH_2_ (30%/163%), TPP-6-YrFK-NH_2_ (97%/122%) (**Figure 5C**).

## Discussion

The tetrapeptides with alternating aromatic and cationic units, i.e., YRFK based motifs, were used in our study as model antioxidant peptides, in which tyrosine or its analog 2′,6′-dimethyltyrosine are considered the main radical-scavenging center irrespective of its position in the peptide sequence (Szeto, 2006a). The aromatic-cationic structure provides increased bioavailability and cytoprotective activity for these tetrapeptides in comparison with other hydrophilic oligopeptides lacking cell-penetrating signals (Zhao et al., 2003; Szeto, 2006b). The effect of modification of the tetrapeptides with DLC, e.g., the TPP cation, on their properties has not been evaluated to date.

YRFK-NH_2_, TPP-3-YRFK-NH_2_, TPP-6-YRFK-NH_2_, YrFK -NH_2_, TPP-6-YrFK-NH_2_ oligopeptides (**Figure 1**) were synthesized and compared to establish proof of concept of a pronounced modulation of antioxidant/cytoprotective properties and proteolytic stability of the modified oligopeptides by the TPP moiety.

According to the DPPH assay, the oligopeptides showed the lack of scavenging capacity against the DPPH-radical, unlike water soluble antioxidants, such as thiols and ascorbic acid (data not shown). Hence, we used sensitive fluorescent assay with the DCFDA probe which relies on Fenton-like CoCl_2_/H_2_O_2_ reaction (Akhmadishina et al., 2017). DCFDA is a diacetylated derivative of 2′,7′-dichlorodihydrofluorescein (2′,7′-dichlorofluorescin), and these are the known probe for different reactive oxygen and nitrogen species (Hempel et al., 1999; Gomes et al., 2005; Rhee et al., 2010). It is considered that the oxidation of the dichlorofluorescin derivatives by H_2_O_2_ is mediated by enzymes and transition metals (Gomes et al., 2005; Rhee et al., 2010), and this is in accordance with our observation that DCFDA oxidation by an excess of H_2_O_2_ is limited by the transition metals which catalyze generation of ROS, such as the hydroxyl radical (Akhmadishina et al., 2017). Among different metals, the cobalt was previously selected as an established prooxidant, which provided the most intense and reproducible ROS generation (Akhmadishina et al., 2017). The initial YRFK based motifs as well as the TPP-modified oligopeptides promoted the CoCl_2_/H_2_O_2_ reaction, mainly, at their micromolar concentrations, however, this promoting effect decreased and changed to ROS suppression with increase in concentration of the oligopeptides (**Figure 3**).

The prooxidant CoCl_2_/H_2_O_2_ reaction was shown to be sensitive to the nature of surrounding solutes, i.e., strongly promoted by phosphate ions and affected by the ability of antioxidants to interact with the metal and, at increased concentrations, directly scavenge ROS (Akhmadishina et al., 2017). Given this, a complex formation between the cobalt ions and certain centers in the tetrapeptides, probably, arginine and lysine residues (Brookes and Pettit, 1976), seems to mediate the Fenton reaction, whereas gradual decrease in ROS formation in the presence of the oligopeptides (TPP-6-YRFK-NH_2_, TPP-3-YRFK-NH_2_, YRFK-NH_2_) at concentrations over 100 μM (**Figure 3**) should be attributed to their antiradical action.

Both pro- and antioxidant activity of the oligopeptides strongly depended on the structure of peptide and TPP components. Unlike YRFK-NH_2_, the YrFK-NH_2_ counterpart was characterized by the lack of antioxidant/decreased prooxidant effects, which were, moreover, slightly affected by the TPP group. Three oligopeptides possessed increased antioxidant properties, namely, YRFK-NH_2_ < TPP-3-YRFK-NH_2_ < TPP-6-YRFK-NH_2_ (**Figure 3**), where the latter modified oligopeptide had comparable EC_50_ value to that of GSH, the predominant antioxidant oligopeptide in mammalian cells. This shows that the TPP moiety enhances the ability of modified oligopeptides to suppress the Fenton reaction, hypothetically, due to combined antiradical action of the TPP cation and tyrosine residue. The enhancing effect is more pronounced for the TPP-6 group with extended alkyl linker (**Figure 3B**).

These results correlate with conformational differences in the oligopeptides as revealed by NMR spectroscopy study (unpublished data). According to the NMR data, the unmodified YRFK-NH_2_ tetrapeptide has a relatively disorganized and mobile structure, whereas YrFK-NH_2_ has bent and more compact structure. Such organization of the latter D-arginine-containing tetrapeptide seems to decrease its reactivity in the Fenton reaction irrespective of the addition of TPP-6 group (**Figure 3**). The spatial structure of YRFK-NH_2_ is stabilized by both the TPP-3 and TPP-6 groups. The latter moiety, however, is characterized by higher flexibility, which seemingly contributes to the increased antioxidant activity of TPP-6-YRFK-NH_2_ (**Figure 3**).

The availability of oligopeptides as cellular antioxidants was initially studied on neuronal PC-12 cells subjected to acute oxidative stress in HBSS (**Figure 4**). All synthesized oligopeptides induced inhibition of H_2_O_2_-driven production of cytosolic ROS only above a threshold level over 1 mM, while completely suppressed cell oxidation at a concentration of 10 mM in a similar manner with GSH. In the case of the oligopeptides, the concentration dependence seemingly does not reflect their limited diffusion into cells. According to (Zhao et al., 2003; Szeto et al., 2005), Dmt-^D^Arg-Phe-Lys-NH_2_ and its fluorescent analogs readily diffuse across plasma membrane both in and out of the cells in a concentration-dependent manner (1 μM–3 mM), and do not rely on energy-dependent mechanisms, such as peptide transporters, opioid receptors and *P*-glycoprotein. Hence, a relatively high intracellular level of the oligopeptides is apparently required to exert antioxidant effect under experimental conditions. In the case of GSH for the same effect, the extracellular concentration of tripeptide should obviously exceed its physiological intracellular level [1–11 mM (Schafer and Buettner, 2001)].

According to the literature data, the exogenic GSH can enter mammalian cells at micromolar/submillimolar concentrations predominantly via different transporters with a relatively high Michaelis constant (Bachhawat et al., 2013). Increased millimolar concentrations of GSH, however, could hypothetically promote its transmembrane diffusion and/or redox reactions with certain freely diffusing cellular mediators to effectively inhibit the oxidative stress (**Figure 4**). The cell-directed antioxidant activity of the oligopeptides (5 mM) increased as follows: TPP-3-YRFK-NH_2_ ≈ YRFK-NH_2_ ≈ YrFK-NH_2_ < GSH < TPP-6-YRFK-NH_2_ ≈ TPP-6-YrFK-NH_2_ (**Figure 4**). These results suggest a potentially high ability of the TPP-modified oligopeptides to diminish acute formation of cytosolic ROS. The TPP-6 moiety augments this activity in a more efficient way than TPP-3 and irrespective of the conformation of oligopeptides, i.e., YRFK-NH_2_ or YrFK-NH_2_ variants. This could be attributed to better membrane binding and translocation of the oligopeptides coupled with the TPP group with a relatively extended aliphatic linker (i.e., TPP-6) in accordance with reported mitochondrial uptake of alkyl-TPP based compounds (Asin-Cayuela et al., 2004; Murphy and Smith, 2007; Khailova et al., 2015).

Our data suggest that modification with the TPP cation allows for the increase in availability (activity) of antioxidant oligopeptides, e.g., YRFK based motifs, at cellular level, though they are initially characterized by high membrane permeability (Zhao et al., 2003; Szeto et al., 2005). The mechanism of ROS-specific activity of the TPP-modified oligopeptides including their potential effect on mitochondrial ROS production will be examined elsewhere.

The MTT assay generally revealed the lack of cytotoxicity of the oligopeptides toward PC-12 cells and HSF upon 3 days of culturing in the concentration range from 1 μM to 3.2 mM (Supplementary Figure S2). An obvious modulating effect of the oligopeptides on proliferation, however, appeared depending on both peptide structure and cell type. In particular, a parabolic-like decrease in HSF viability was observed with a minimum at ∼100 μM concentration of the oligopeptides. Such effect was more pronounced for the TPP-modified oligopeptides seemingly due to their increased cellular availability and/or antioxidant activity. Furthermore, TPP-3-YRFK-NH_2_ and TPP-6-YRFK-NH_2_ significantly stimulated proliferation of PC-12 cells in a wide concentration range (Supplementary Figure S2).

These effects could be explained by the ability of oligopeptides to modulate intracellular ROS and ROS-dependent redox balance. At a certain level, ROS (e.g., mediated by Fenton reactions) are known to reversibly activate mitogenic signals in mammalian cells, including fibroblasts, and the elimination of these ROS causes a decrease in cell proliferation (Kim et al., 2001). The oligopeptides are expected to interfere with ROS-mediated proliferation of the HSF at certain concentrations, while diminish elevated ROS level in PC-12 cells, which is typical for cancer cell metabolism (Liou and Storz, 2010), resulting in opposite cell-stimulating effect (Supplementary Figure S2). Such effect, however, seems to be cell-specific, since the oligopeptides did not affect viability of prostate cancer (PC-3) cells (data not shown), unlike GSH which greatly stimulated proliferation of PC-3 cells under the same supplementation (Akhmadishina et al., 2017).

Neuroprotective potential of the modified oligopeptides was evaluated using PC-12 cells as a relevant *in vitro* model (Zou et al., 2001; Lee et al., 2004; Penugonda et al., 2005; Tripathy and Grammas, 2009). The cells were exposed to several agents that induce oxidative damage and apoptosis in mammalian cells through different mechanisms. In particular, CoCl_2_ was used to mimic hypoxic responses in cells via ROS generation and up-regulation of related transcriptional factors (Salnikow et al., 2000; Zou et al., 2001). At millimolar concentrations ^L^Glu causes receptor-independent oxidative stress and apoptosis in PC-12 cells which involve mitochondria depolarization, GSH depletion and elevation of cytosolic calcium level (Pereira and Oliveira, 2000; Lee et al., 2004; Penugonda et al., 2005). Furthermore, PC-12 cells were treated with Men, a quinone compound which mediates formation of superoxide radical and exhibits pro-inflammatory and apoptotic activities (Tripathy and Grammas, 2009). To escape strong and varying protective effect of FBS, serum-free medium was used for the investigation.

Cell response to the above compounds differed depending on the damaging factor and whether the oligopeptides and factors were supplemented into the medium sequentially or jointly. The lack of protective effect of the pre-added YRFK based oligopeptides, both the unmodified ones and TPP derivatives, on CoCl_2_-treated cells was observed, whereas coadministration of the compounds resulted in a significant increase in cell viability (**Figure 5A**). The cytoprotection provided by the modified oligopeptides, i.e., TPP-3-YRFK-NH_2_ and TPP-6-YRFK-NH_2_, was much more pronounced.

These results show that the ability of YRFK based motifs to diminish ROS overproduction by transition metals *in vitro* could be noticeably enhanced by conjugation with the TPP moiety. Furthermore, considerable increase in viability of the cells treated with TPP-3-YRFK-NH_2_ and TPP-6-YRFK-NH_2_ to exceed control values (untreated cells) (**Figure 5A**) suggests that the oligopeptides exhibit noticeable supporting and/or stimulating effect on metabolic/proliferative activity of the cells in FBS-free medium. The fact that the cytoprotective effect of oligopeptides was achieved only upon their coaction with CoCl_2_ (**Figure 5A**) indicates a relatively fast clearance of the oligopeptides presumably as a result of their efflux out of the cells and biodegradation.

Similar relationships were revealed for ^L^Glu-treated PC-12 cells, which were effectively protected and stimulated by TPP-3-YRFK-NH_2_, TPP-6-YRFK-NH_2_ and TPP-6-YrFK-NH_2_ only when added simultaneously with ^L^Glu (**Figure 5B**). This suggests a somewhat similar damaging action of ^L^Glu and CoCl_2_ on PC-12 cells and also supports enhanced cytoprotective effect of the TPP-modified oligopeptides against the prooxidant factors over the unmodified tetrapeptides. Interestingly, only TPP-6-YrFK-NH_2_ completely prevented cell damage by ^L^Glu even when supplemented 24 h prior to the damage (**Figure 5B**), seemingly due to increased proteolytic resistance of this oligopeptide (**Figure 2B**).

In contrast to the modified oligopeptides, GSH slightly affected viability of PC-12 cells treated with CoCl_2_ and ^L^Glu (**Figures 5A,B**). The lack of effect of GSH could be attributed to its insufficient cellular transportation as well as strong regulation of the intracellular GSSG/GSH ratio which restrict antioxidative/cytoprotective potential of the tripeptide under these particular conditions.

The treatment of PC-12 cells with Men was shortened to 3 h due to its increased toxicity, and therefore, in addition to above two ways of administration, after exposure to Men the cells were also cultured in the presence of oligopeptides for 24 h (without Men). The Men-treated cells responded to the oligopeptides in a distinct manner from the cells exposed to CoCl_2_ and ^L^Glu. In particular, GSH noticeably decreased Men-induced cytotoxicity when applied together with or, in less extent, followed Men action (**Figure 5C**) in accordance with evidence that this quinone depletes GSH in non-resistant cells (Chiou and Tzeng, 2000). The unmodified YRFK and YrFK motifs increased viability of Men-treated cells to values similar to those of GSH, but only when applied before and after the treatment. Upon cotreatment with Men, these tetrapeptides, unlike the TPP-modified variants and GSH, promoted Men cytotoxicity (**Figure 5C**).

Furthermore, both pre- and cotreatment of PC-12 cells with the TPP-modified tetrapeptides were accompanied by similar restoration of cell viability, and their activity increased as follows: TPP-6-YRFK-NH_2_ < TPP-3-YRFK-NH_2_ < TPP-6-YrFK-NH_2_. Following Men action, subsequent 24 h culturing of the cells with the oligopeptides prevented cell damage and further increased cell viability over control (**Figure 5C**, posttreatment) in a similar manner as observed for other damaging factors (**Figures 5A,B**, cotreatment). Our results show usefulness of the comparative analysis of PC-12 cells treated in different conditions for establishing cytoprotective properties of antioxidant oligopeptides.

In addition, we studied the effect of TPP moiety on proteolytic resistance of the oligopeptides, which was also considered relative to their cytoprotective activity. Intense enzymatic degradation of peptides, which are typically characterized by a short half-life within a few minutes, is one of the main obstacles to developing peptide based therapeutics (Adessi and Soto, 2002). Masking effects of amino acid isomers and analogs as well as different N- and C-termini modifications against recognition and cleavage by exo- and endopeptidases are well documented (Adessi and Soto, 2002; Hamamoto et al., 2002; Tugyi et al., 2005; Meng and Kumar, 2007; Gentilucci et al., 2010; Vlieghe et al., 2010; Danial et al., 2012).

Regarding YRFK based motifs, introduction of D-arginine instead of the L-enantiomer was reported to render these tetrapeptides protease-resistant (Szeto, 2006a). In accordance with these data, YrFK-NH_2_ was characterized by approximately 3.5-fold slower tryptic cleavage than that of YRFK-NH_2_ as revealed by reaction time required for 50%-degradation (**Figure 2**). The opposite effect of the TPP moiety on proteolytic stability of these tetrapeptide variants was unexpectedly observed. In particular, both the TPP-3 and TPP-6 groups substantially promoted the proteolytic reaction with YRFK-NH_2_, though the corresponding modified oligopeptides exhibited somewhat different kinetics (**Figure 2A**). This promotion could be explained by more arranged structure of the oligopeptides coupled with the TPP unit, which probably contributes to trypsin endopeptidase activity. Moreover, lipophilic and cationic structure of the TPP moiety might hypothetically mediate interaction of the modified oligopeptides with hydrophobic and anionic sites in the active center of trypsin which are located in proximity to the catalytic site (Mares-Guia et al., 1967). We believe that the delay in TPP-6-YRFK-NH_2_ cleavage (**Figure 2A**) results from aggregative properties of the oligopeptide, which retard its interaction with the enzyme at the initial stage of the reaction.

The tolerance of TPP-6-YrFK-NH_2_ to trypsin cleavage could be further explained by its conformational features according to NMR spectroscopy study (unpublished data). Specifically, due to the bent backbone and more compact structure of D-arginine-containing YrFK, the most probable cleavage site between arginine and phenylalanine residues (Olsen et al., 2004; Vlieghe et al., 2010) is less available for cleavage. The TPP moiety provides an additional obstacle to the site, apparently, making it even less available for trypsinolysis. These primary results suggest the route of development of protease-resistant therapeutic peptides by introducing D-amino acids and the TPP moiety into a sequence near to cleavage sites.

The correlation between proteolytic stability of the oligopeptides (**Figure 2**) and their cytoprotective activity (**Figure 5**) is difficult to establish, as the latter activity should depend on different factors, at least, the type of oxidative agent, mode of treatment, cellular pharmacokinetics and antioxidant efficacy. Enhanced cytoprotection exhibited by the TPP-modified oligopeptides seemingly results from their better cellular availability and antioxidant effect (**Figures 3**, **4**), whilst the most active oligopeptides (TPP-3-YRFK-NH_2_ and TPP-6-YRFK-NH_2_) are readily degraded (**Figure 2A**). The proteolytic stability of TPP-6-YrFK-NH_2_, however, contributes to its cytoprotective activity, as only this oligopeptide effectively prevented cell damage by ^L^Glu and Men when administered 24 h prior to the damaging agents (**Figures 5B,C**).

Altogether, our results demonstrate the potential of DLC in regulation of pharmacokinetic and pharmacological properties of small bioactive peptides. Solid-phase peptide synthesis provides a controllable introduction of DLC derivatives into peptide sequences. We have shown that the modification of YRFK sequence motifs with proved antiapoptotic and neuroprotective properties (Szeto, 2006a,b, 2008) with the carboxyalkyl-TPP units allows for noticeable enhancement of the activity of oligopeptides *in vitro*. The TPP moiety potentiates the protective effect of YRFK based tetrapeptides on neuronal PC-12 cells against different oxidative factors, which mimic acute and chronic damage in neuronal tissues (**Figure 5**). Given the potentiating effect of TPP moiety together with its ability to impart the proteolytic stability to oligopeptides (**Figure 2B**) and established carrier properties (Murphy and Smith, 2007), the TPP-modified antioxidant oligopeptides can be regarded as promising candidates for therapeutic peptides. Further investigation of the bioactivity of TPP-modified oligopeptides toward specific ROS and ROS-associated diseases will be performed elsewhere using relevant pharmacological models.

## Author Contributions

RA performed *in vitro* study. RG synthesized and verified the oligopeptides. NP studied proteolytic stability. MK performed physicochemical characterization. TA designed the research and wrote the manuscript. All authors read and approved the final manuscript.

## Conflict of Interest Statement

The authors declare that the research was conducted in the absence of any commercial or financial relationships that could be construed as a potential conflict of interest.
