# Supplementary material for: Triphenylphosphonium Moiety Modulates Proteolytic Stability and Potentiates Neuroprotective Activity of Antioxidant Tetrapeptides in Vitro
Source: Front Pharmacol. 2018 Feb 19;9:115. doi: 10.3389/fphar.2018.00115 (PMC5827532; doi:10.3389/fphar.2018.00115)
Supplement: Supplementary file 1 [file Presentation_1.pdf]

SUPPLEMENTARY MATERIAL

YRFK-NH$_2$

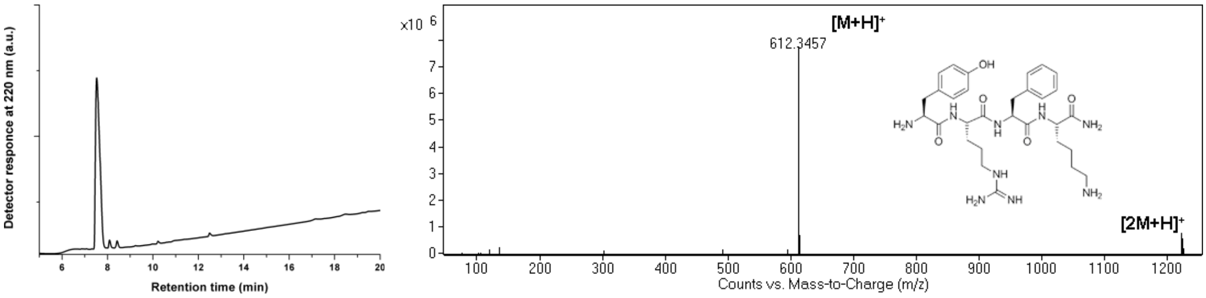

TPP-3-YRFK-NH$_2$

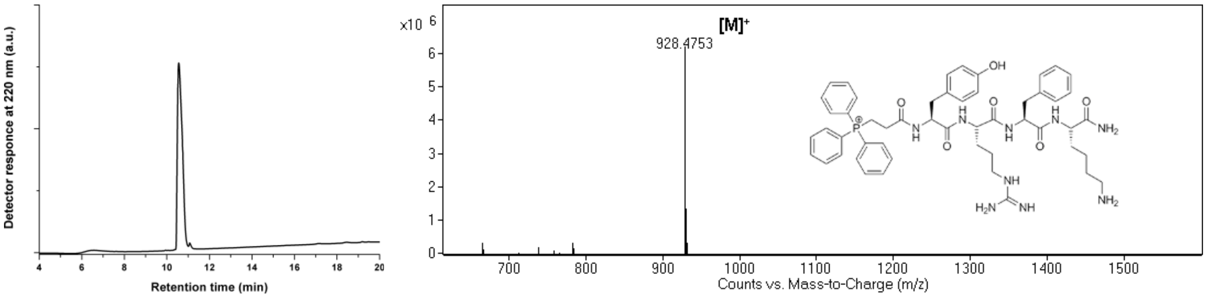

TPP-6-YRFK-NH$_2$

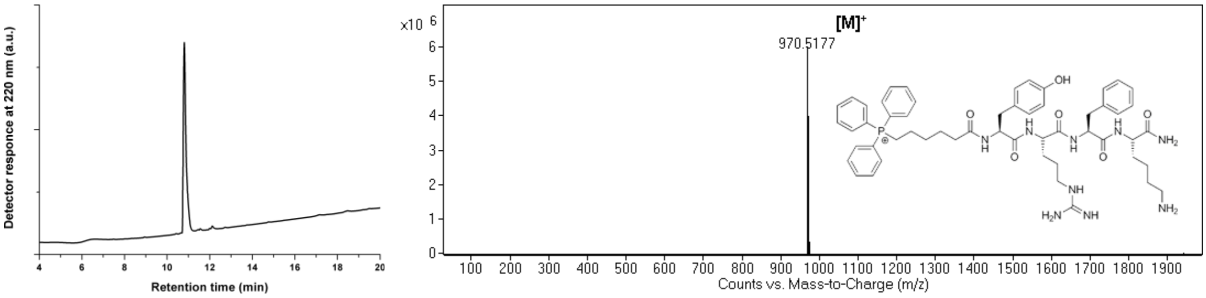

YrFK-NH$_2$

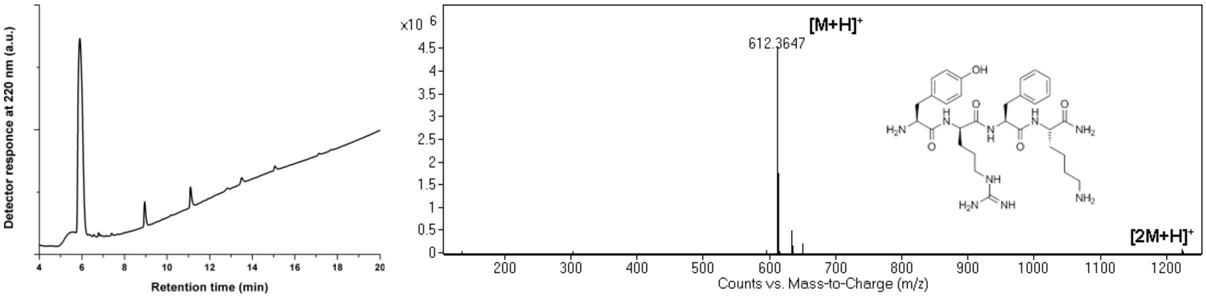

TPP-6-YrFK-NH$_2$

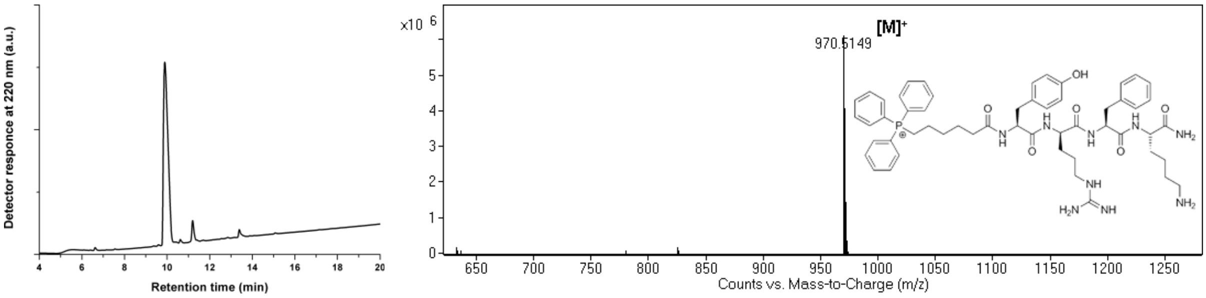

FIGURE S1. LC-MS spectra of YRFK based tetrapeptides and their carboxyalkyl triphenylphosphonium derivatives.

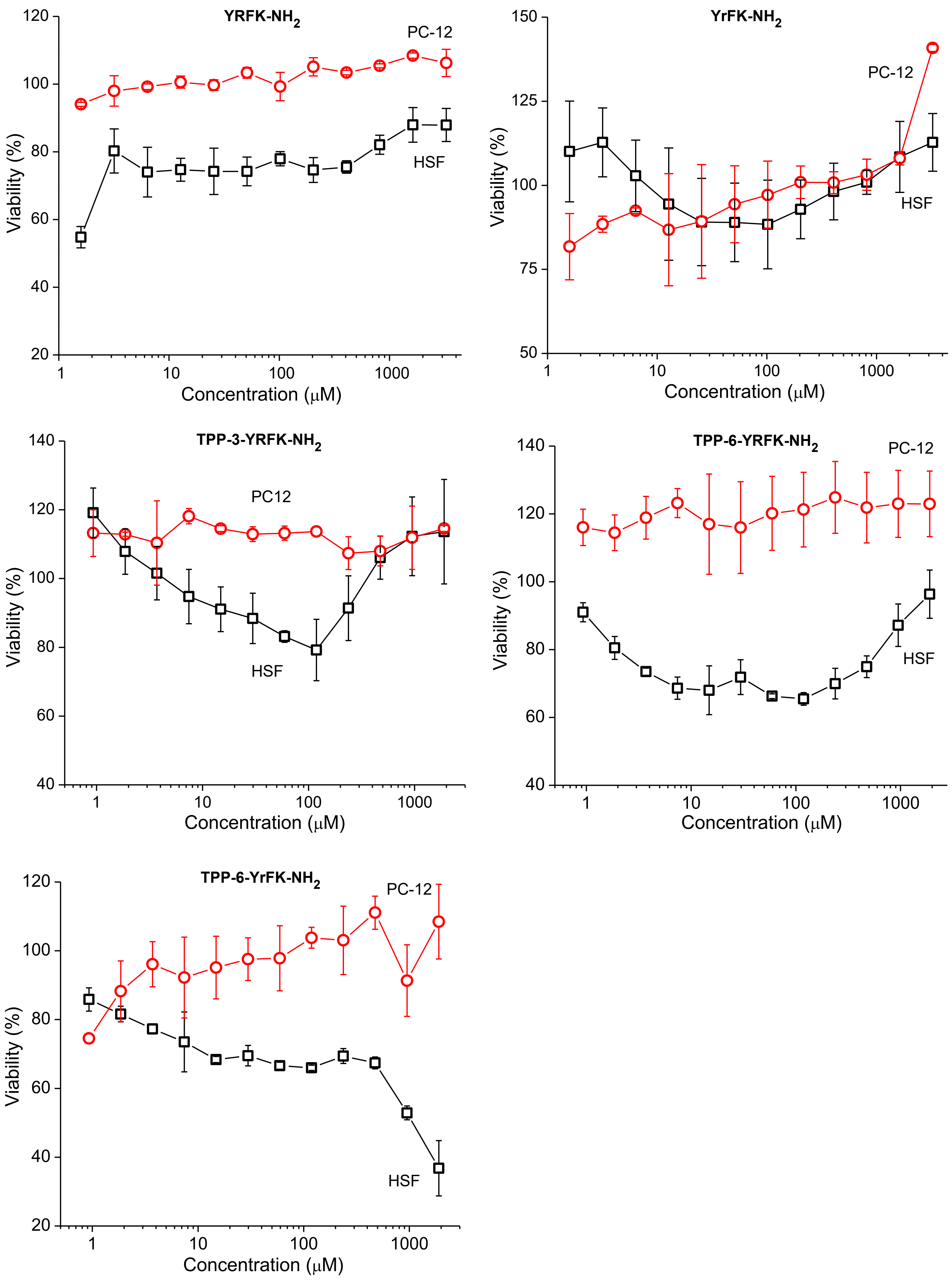

Figure S2. Concentration-cell viability curves of YRFK based tetrapeptides and TPP-derivatives for PC-12 cells and human skin fibroblasts (HSF). MTT-assay (72 h).
